# Supplementary material for: Modeling glioblastoma heterogeneity as a dynamic network of cell states
Source: Mol Syst Biol. 2021 Sep 16;17(9):e10105. doi: 10.15252/msb.202010105 (PMC8444284; doi:10.15252/msb.202010105)
Supplement: Supplementary file 5 — Source Data for Figure 3 [file MSB-17-e10105-s001.zip › Figure3A_sourcedata/GSEA_3065/nosigdb_state2.GseaPreranked.1622718419215/OLSEN_MESENCHYMAL.html]

Details for gene set OLSEN\_MESENCHYMAL[GSEA]

|  || Dataset | state2 |
| Phenotype | NoPhenotypeAvailable |
| Upregulated in class | na\_neg |
| GeneSet | OLSEN\_MESENCHYMAL |
| Enrichment Score (ES) | -0.7511445 |
| Normalized Enrichment Score (NES) | -2.671261 |
| Nominal p-value | 0.0 |
| FDR q-value | 0.0 |
| FWER p-Value | 0.0 |
Table: GSEA Results Summary

  

Fig 1: Enrichment plot: OLSEN\_MESENCHYMAL      
 Profile of the Running ES Score & Positions of GeneSet Members on the Rank Ordered List

  

| PROBE | GENE SYMBOL | GENE\_TITLE | RANK IN GENE LIST | RANK METRIC SCORE | RUNNING ES | CORE ENRICHMENT || 1 | RPL41 |  |  | 313 | 0.227 | -0.0148 | No |
| 2 | RPS25 |  |  | 413 | 0.197 | -0.0092 | No |
| 3 | RPLP1 |  |  | 648 | 0.156 | -0.0213 | No |
| 4 | RPL32 |  |  | 661 | 0.155 | -0.0099 | No |
| 5 | MT2A |  |  | 731 | 0.146 | -0.0053 | No |
| 6 | EEF1D |  |  | 783 | 0.140 | 0.0008 | No |
| 7 | EGR1 |  |  | 1691 | 0.077 | -0.0896 | No |
| 8 | SERF2 |  |  | 1979 | 0.065 | -0.1149 | No |
| 9 | ZFP36L1 |  |  | 2320 | 0.053 | -0.1468 | No |
| 10 | LY6E |  |  | 2588 | 0.045 | -0.1716 | No |
| 11 | LIMA1 |  |  | 3279 | 0.028 | -0.2430 | No |
| 12 | JUNB |  |  | 3923 | 0.017 | -0.3102 | No |
| 13 | UACA |  |  | 3925 | 0.017 | -0.3089 | No |
| 14 | TPT1 |  |  | 4568 | 0.006 | -0.3769 | No |
| 15 | NNMT |  |  | 5544 | -0.009 | -0.4802 | No |
| 16 | S100A11 |  |  | 5569 | -0.009 | -0.4820 | No |
| 17 | CCDC80 |  |  | 6077 | -0.018 | -0.5347 | No |
| 18 | IFITM2 |  |  | 6128 | -0.019 | -0.5385 | No |
| 19 | LHFPL6 |  |  | 6336 | -0.022 | -0.5587 | No |
| 20 | AEBP1 |  |  | 7162 | -0.041 | -0.6434 | No |
| 21 | FOS |  |  | 7222 | -0.043 | -0.6462 | No |
| 22 | ANXA1 |  |  | 7265 | -0.044 | -0.6471 | No |
| 23 | TIMP3 |  |  | 7309 | -0.045 | -0.6480 | No |
| 24 | FTL |  |  | 7693 | -0.056 | -0.6842 | No |
| 25 | TPM2 |  |  | 8019 | -0.068 | -0.7133 | No |
| 26 | THY1 |  |  | 8139 | -0.074 | -0.7200 | No |
| 27 | NPC2 |  |  | 8297 | -0.083 | -0.7300 | No |
| 28 | TMSB4X |  |  | 8392 | -0.089 | -0.7328 | No |
| 29 | PRRX1 |  |  | 8427 | -0.091 | -0.7290 | No |
| 30 | LMNA |  |  | 8636 | -0.106 | -0.7425 | Yes |
| 31 | C1R |  |  | 8644 | -0.107 | -0.7345 | Yes |
| 32 | TAGLN |  |  | 8662 | -0.108 | -0.7275 | Yes |
| 33 | LGALS1 |  |  | 8680 | -0.110 | -0.7203 | Yes |
| 34 | PCOLCE |  |  | 8685 | -0.110 | -0.7117 | Yes |
| 35 | VIM |  |  | 8691 | -0.110 | -0.7032 | Yes |
| 36 | CST3 |  |  | 8712 | -0.112 | -0.6961 | Yes |
| 37 | IFITM3 |  |  | 8720 | -0.113 | -0.6876 | Yes |
| 38 | SELENOM |  |  | 8855 | -0.129 | -0.6913 | Yes |
| 39 | S100A13 |  |  | 8880 | -0.133 | -0.6830 | Yes |
| 40 | PRSS23 |  |  | 8975 | -0.145 | -0.6811 | Yes |
| 41 | SERPINH1 |  |  | 9054 | -0.159 | -0.6764 | Yes |
| 42 | COL4A2 |  |  | 9069 | -0.161 | -0.6647 | Yes |
| 43 | CAVIN1 |  |  | 9071 | -0.161 | -0.6516 | Yes |
| 44 | S100A6 |  |  | 9097 | -0.166 | -0.6407 | Yes |
| 45 | COL6A2 |  |  | 9116 | -0.170 | -0.6287 | Yes |
| 46 | PPIC |  |  | 9122 | -0.171 | -0.6152 | Yes |
| 47 | COL5A1 |  |  | 9148 | -0.177 | -0.6034 | Yes |
| 48 | EMP1 |  |  | 9156 | -0.179 | -0.5895 | Yes |
| 49 | FSTL1 |  |  | 9158 | -0.179 | -0.5749 | Yes |
| 50 | CD99 |  |  | 9224 | -0.203 | -0.5652 | Yes |
| 51 | GSN |  |  | 9238 | -0.206 | -0.5497 | Yes |
| 52 | ID3 |  |  | 9252 | -0.214 | -0.5336 | Yes |
| 53 | COL4A1 |  |  | 9284 | -0.226 | -0.5184 | Yes |
| 54 | RRBP1 |  |  | 9287 | -0.227 | -0.4999 | Yes |
| 55 | IGFBP7 |  |  | 9295 | -0.229 | -0.4819 | Yes |
| 56 | COL5A2 |  |  | 9316 | -0.244 | -0.4641 | Yes |
| 57 | COL14A1 |  |  | 9327 | -0.250 | -0.4446 | Yes |
| 58 | FN1 |  |  | 9364 | -0.294 | -0.4243 | Yes |
| 59 | B2M |  |  | 9383 | -0.331 | -0.3991 | Yes |
| 60 | SPARC |  |  | 9384 | -0.332 | -0.3719 | Yes |
| 61 | TIMP1 |  |  | 9387 | -0.334 | -0.3447 | Yes |
| 62 | CALD1 |  |  | 9391 | -0.348 | -0.3165 | Yes |
| 63 | CD63 |  |  | 9394 | -0.353 | -0.2877 | Yes |
| 64 | VCAN |  |  | 9396 | -0.364 | -0.2580 | Yes |
| 65 | ITM2B |  |  | 9399 | -0.371 | -0.2278 | Yes |
| 66 | COL6A1 |  |  | 9410 | -0.410 | -0.1953 | Yes |
| 67 | POSTN |  |  | 9437 | -0.750 | -0.1366 | Yes |
| 68 | IGFBP5 |  |  | 9438 | -0.797 | -0.0713 | Yes |
| 69 | NEAT1 |  |  | 9439 | -0.871 | 0.0001 | Yes |
Table: GSEA details [plain text format]

  

Fig 2: OLSEN\_MESENCHYMAL: Random ES distribution      
 Gene set null distribution of ES for **OLSEN\_MESENCHYMAL**
